# Preliminary study between Y chromosome haplogroups and chagasic cardiomyopathy manifestations in patients with Chagas disease

**DOI:** 10.1590/0037-8682-0566-2019

**Published:** 2020-11-06

**Authors:** Oscar Lassen, Sandra Tabares, Patricia Bertolotto, Silvia Ojeda, Adela Sembaj

**Affiliations:** 1 Cordoba Hospital, Semiology Department UHMI 3, Chagas and Hypertension Office, Córdoba, Argentina.; 2 School of Medicine, Biochemistry and Molecular Biology Department, UNC, Cordoba, Argentina.; 3 School of Mathematics, Astronomy and Physics, UNC, Córdoba, Argentina.

**Keywords:** Chagas Disease, Cardiomyopathy, Y chromosome

## Abstract

**INTRODUCTION:**

Among patients with Chagas disease, men have a higher risk of worse pathological symptoms than women. We aimed to explore the role of the Y chromosome in men diagnosed with Chagas disease and assess the relationship between their ancestry and disease status.

**METHODS:**

In this comparative study, we analyzed 150 men with unrelated non-chagasic disease (nCD) and 150 men with unrelated chagasic disease (CD). We assessed the serological diagnosis of Chagas disease, biochemical parameters, thoracic X-rays, electrocardiogram, and transthoracic echocardiography and determined the haplogroup by analyzing a set of 17 microsatellites from the Y chromosome. We examined the associations between common Y chromosome haplogroups and the clinical parameters of risk by logistic regression.

**RESULTS:**

For all patients, the most common haplogroups were R1b (43%), G2a (9%), and E1b1b (9%). The R1b and G2a haplogroup was more frequent in men with nCD and CD, respectively. As expected, we observed a high proportion of symptomatic patients in the CD group independent of the haplogroups. Men from both groups classified as having the R1b haplogroup showed less clinical evidence of disease. Multivariate analysis showed that CD patients without R1b were about five times more likely to have a cardio-thorax index >0.5% (OR [odds ratio] = 5.1, 95% CI [confidence interval] = 3.31-8.17). Men without the R1b haplogroup were 2.5 times more likely to show EcoCG alterations (OR = 2.50, 95% CI = 0.16-3.94).

**CONCLUSIONS:**

Our results provided evidence that the R1b haplogroup may have a potential protective cardiovascular effect for its carriers.

## INTRODUCTION

Chagas disease (CD) is a parasitic infection caused by the protozoa *Trypanosome cruzi (T. cruzi)*. This disease affects millions of people in South and Central America^1^. Parasites enter the blood flow and infect a wide variety of cells, which leads to the initial stage of acute infection characterized by high parasitemia and tissue parasitism^2^. After the acute stage, patients enter the chronic stage, which is characterized by the lifelong presence of circulating anti-*T. cruzi* antibodies. This stage is also characterized by a large difference in clinical indicators from those associated with an asymptomatic phenotype, such as severe cardiac and gastrointestinal damage^2^. Approximately 30% of the infected chronic patients develop chronic chagasic cardiomyopathy (CCC)^3^ ^,^ ^4^. CCC is one of the main causes of congestive heart failure in Latin America^5^ ^,^ ^6^. It involves complex arrhythmia, heart block, and thromboembolic events, among other affections^5^ ^,^ ^6^. 

Currently, many uncertainties exist regarding the pathophysiological mechanisms involved in CCC development and it is not known why certain patients from endemic areas survive without symptoms, while others develop severe arrhythmia or suffer sudden death at a certain point in their lives^3^. Clinical progression, survival rate, and the global projection are significantly worse in CCC patients than in patients with other non-inflammatory dilated cardiomyopathies^6^. Regarding CD, Pereira et al. found that the evolution of CCC was associated with the age and gender of the patients^7^. It was shown that men presented with significantly higher progression risk of CCC than women^8^.

Several studies in the general population reported relationships between polymorphisms of a single nucleotide in the male-specific region of the Y chromosome (MSY) and blood pressure, total plasma level of cholesterol, low density lipoprotein cholesterol, and male parent history of myocardial arrest and coronary diseases^9^. Moreover, it was shown that such conditions associated with the Y chromosome are significantly overexpressed in patients with idiopathic dilated cardiomyopathy. Such evidence supports the idea that gene variation within the MSY may have an important role in determining cardiovascular risks in men. Charchar et al. showed that British men who inherited haplogroup I from their male ancestors had a 50% higher risk of developing acute coronary diseases compared to men with other Y chromosome haplogroups^9^.

The aim of this study was to analyze the frequency of haplogroups of the Y chromosome in a sample of patients infected with *T. cruzi* and to establish a possible relationship between haplogroups of the Y chromosome and the clinical cardiovascular characteristics of CD.

## METHODS

In this case-control study, 300 unrelated men who consecutively attended the Hypertension Department of the Córdoba Hospital in the city of Córdoba, Argentina were randomly selected. Every patient completed a form on their family medical histories of cardiovascular disease, and other current diseases. A physical assessment was carried out according to the ethical rules (Helsinki-Armonization) and informed consent was acquired from the patients before admission. The study was approved by the Institution Ethics Committee (RePIS 2517). Patients with the following characteristics were excluded: minor patients (under the age of 21), secondary hypertension, diabetes mellitus, active malign neoplastic conditions, drug or alcohol abuse, previous bone marrow allogeneic transplant, whole blood transfusion with no reduction in white blood cells within 120 days of the blood test, patients on treatment regimens with corticoids, immunosuppressors and/or chronic non-steroidal anti-inflammatory drugs. 

After the physical assessment was performed, a 12-lead electrocardiogram (ECG) at rest was carried out (Fukuda Denshi FX 7402 Cardimax) at the Cardiology and Radiology Department of the Hospital of Córdoba, a transthoracic echocardiography (TTE) in M-mode, 2D, color, with pulsed and continuous-wave Doppler, and multifrequency transducer (Toshiba Medical Systems- SSA-580a), and a thoracic X-ray (XR) (Siemens 1000 A-Polidoros IT). Results of the TTE, ECG, and thoracic XR were reported by professional practitioners^10^ ^,^ ^11^ ^,^ ^12^. A blood sample analysis was performed on each patient at the central Hospital Laboratory to evaluate the hepatic and kidney functions, plasmatic pattern of lipids, metabolic profiles, and Y chromosome molecular composition. Serological diagnosis of CD was based on enzyme-linked immunosorbent assay (ELISA) and indirect hemagglutination test, according to the World Health Organization (WHO) recommendations^13^.

Systolic blood pressure (SBP) and diastolic blood pressure (DBP) were measured using a standard mercury blood pressure gauge. SBP>140 mmHg, DBP>90 mmHg, or both were considered as thresholds for the diagnosis of hypertension as per the criteria of the WHO and International Society of Hypertension. The cardiothoracic index (CTI) was analyzed using conventional methods, and enlarged heart was defined as CTI>0.5%^14^. The following definitions were adjusted to the American Society of Electrocardiogram guidelines: atrial fibrillation, complete right bundle branch block, complete left bundle branch block, atrioventricular block, ventricular tachycardia, and ventricular fibrillation among others^11^. TTE measurements were carried out according to standard protocols. Left ventricle ejection fraction was determined by the *Teichholz* method and as per international regulations^12^.

Individuals analyzed were divided into two groups, with CD (CD group) and without CD (nCD group), according to the serological tests. Subsequently, patients were classified according to the clinical and biochemical tests and non-invasive studies as mildly symptomatic or asymptomatic patients without cardiac symptoms, and with normal ECG and TTE with some risk factors (hypertension, level of plasmatic cholesterol). The other group included symptomatic or cardiomyopathic patients with different clinical severity conditions who showed one or more of the following characteristics: different conductance alterations (such as complete right bundle branch block), left ventricular hypertrophy, repolarisation disorders and/or complex ventricular arrhythmia, structural cardiomyopathy abnormalities determined by TTE (such as cavity dilations and ventricular dysfunction [diminished ejection fraction]), and cardiomegaly (different heart enlargements) as determined by chest radiography^14^. All the patients resided in the same geographical region, they had been living in the city of Córdoba from more than 10 years, and they shared the same socioeconomic backgrounds and environmental life conditions. The population in this area was homogeneous, which meant that there were no concentrations of other ethnic groups.

### Molecular Analysis of Y Chromosome

Deoxyribonucleic acid (DNA) was extracted from a blood sample mixed with EDTA (Sigma So Louis, USA) and quantified using conventional methods^14^ ^,^ ^15^. DNA was quantified using a spectrophotometer (Beckman Coulter DU 640). DNA samples were adjusted to reach a level of 0.1 ng/µL. Subsequently, 0.5 ng of the sample was amplified using DYS390 marker to assess the optimum concentration of the DNA template in order to generate the Y chromosome haplotypes. The samples were then amplified using Amp FISTRYF filer Kit (Applied Biosystems). The polymerase chain reaction (PCR) products (2 µL) were separated by fluorescence capillary electrophoresis using GA3130 gene analyzer (Applied Biosystems) under standard conditions of 3 KV, a 5-s injection, and an 18,800-s runtime. Data were analyzed using GeneMapper Software v3.1 (Applied Biosystems). Haplogroups of the Y chromosome were defined using the Haplogroup Predictor (http://www.hprg.com/hapest5/)^16^ and were named according to Haplogroup Predictor (ISOGG 2012; http: //www.isogg.org /tree/ISOGG_Y DNA_SNP_Index.html)^17^.

### Statistical Analysis

The statistical analysis was performed using Infostat/P2018 (Argentina). It was established for a random sample of 300 patients as the sample size, with a security coefficient of 95% for a Type I probability error (alpha) of 0.05, with the fact that between 12 and 13 male patients attended the hypertension consulting room of the Córdoba Hospital each week being taken into account.

Data were examined for normality using the Kolmogorov-Smirnov test. The data that did not achieve normality were analyzed using nonparametric methods. Continuous variables were presented as mean values and standard deviations, while categorical and qualitative variables were presented as frequencies (percentages). Comparisons between the continuous variables (age, SBP, DBP, heart rate, weight) were performed using the Student’s t-test for independent samples. Categorical variables were analyzed using the chi-square test or Fisher’s exact test. The relationships between the Y chromosome haplogroups and the clinical parameters for CCC were evaluated using logistic regression, and the corresponding odds ratios (ORs) and 95% confidence intervals (CIs) were determined and used as the response variables of the affected population. For the multivariate analysis, covariates of the presence/absence of a haplogroup and presence/absence of CD were age, cholesterol level, and plasma lipoprotein level. For all the analyses, the significance level was set at 0.05.

## RESULTS

Table 1 shows the data of the biochemical assessments and imaging diagnoses of the participating patients of each group. We did not observe differences in the biochemical plasma parameters between the CD and nCD patients, except for the mean of the plasmatic urea, which was significantly higher in nCD patients (p=0.004). From the thoracic XRs, we observed higher frequencies of alterations in patients with CD (p=0.003) besides patients with heart enlargement grade I, III, and IV; changes in the ECG (with different severities) (p=0.001) and alterations in the TTE (p=0.04) were more frequent in the CD group. 

**TABLE 1:** Biochemical and clinical characteristics of the population at baseline.

	Chagas disease group 150		Non-Chagas disease group 150		*p*
	Mean	SD	Mean	SD	
Age (years)	61	11	56	14	0.555
Glycemia (mg/dL)	95	97	94	96.5	0.782
CPK (U/l)	92	105	90	95	0.358
Urea (mg/dL)	33	9.2	41.6	10.4	**0.004**
Creatinine (mg/dL)	0.89	0.91	0.87	0.89	0.718
Cholesterol (mg/dL)	221	56	214	57	0.415
TG (mg/dL)	156	66	161	83	0.666
HDL Cholesterol (mg/dL)	51.22	10.4	49	12	0.854
LDL Cholesterol (mg/dL)	122	35	117	36	0.526
Thoracic XR					
■ No alterations (0)	19 (12.6%)		32 (21.3%)		**0.045**
■ Heart enlargement grade I	24 (15.9%)		45 (30.0%)		**0.003**
■ Heart enlargement grade II	64 (42.8%)		62 (41.3%)		0.964
■ Heart enlargement grade III and IV	43 (28.6%)		11 (7.0%)		**0.001**
ECG					
■ No alterations	19 (12.6%)		36 (24.0%)		**0.011**
■ CRBBB + LVH	36 (24.0%)		52 (34.6%)		**0.042**
■ + repolarization disorders	88 (58.6%)		61 (40.6%)		**0.018**
■ + other conditions	7 (4.6%)		1 (0.67%)		**0.031**
TTE					
■ No alterations	19 (12.6%)		90 (60.0%)		**0.001**
■ EF 60% + cavity dilation	43 (28.6%)		31 (20.6%)		0.110
■ EF less that 59%	9 (6.0%)		10 (6.6%)		0.811
■ EF less that 59%+cavity dilation	79 (52.6%)		19 (12.0%)		**0.001**

Values are shown as mean and standard deviation (SD); in the cases of frequencies, number of subjects is presented, and percentages are in brackets. Normal values: Glycemia: 70-110 mg/dL; CPK (creatine phosphokinase): 0-170 U/l; Urea: 40 mg/dL; Creatinine: 0.7-1.4 mg/dL; Cholesterol: <200 mg/dL; TG (Triglycerides): <150 mg/dL; HDL-cholesterol (high density lipoprotein cholesterol): >40 mg/dL; LDL-cholesterol (low density lipoprotein cholesterol): <100 mg/dL; CRBBB (complete right bundle branch block); LVH (left ventricular hypertrophy); EF (ejection fraction). p-values in bold letters are statistically significant values. Significance level was p<0.05.

Haplotype diversity (Hd) showed an index of 0.785 for the total population; in particular, Hd_Chagas_ was 0.88 in the CD group, while it was 0.69 in the nCD group, which indicates that both groups had a high level of genetic diversity in the Y chromosome. Table 2 shows the frequencies of the Y chromosome haplogroups in the total population and the study groups. The most representative haplogroups shown in the sample were R1b (41%), G2a (10%), and E1b1b (8%). The comparison of the ratios of the representative haplogroups in the sample between patients with and without CD was performed using Fisher’s exact test (haplogroup G2a, p=0.034; haplogroup R1b, p=0.042; and haplogroup E1b1b, p=0.078). The most frequent haplogroup in the population sample was R1b, although it was more frequent in nCD patients; in CD patients, the most frequent haplogroup was G2a. A comparison of haplogroup R1b and the rest of the haplogroups for each biochemical variable did not show any differences. As shown in Table 2, there were several comparisons with statistical significance; however, there were low number of cases in each haplogroup. Therefore, to obtain representative results, we decided to perform a statistical analysis with only the haplogroups and with a robust sample number. 

**TABLE 2:** Haplogroup frequencies of the Y chromosome in the study groups.

Haplogroups	Chagas group	Non-Chagas group	Total population	p-value
	n=150	n=150	n=300	
	n(%)	n(%)	n(%)	
J2a1b	6 (4)	4 (3)	10 (33)	0.052
G2a	28 (19)	4 (3)	32 (10.6)	**0.001**
J1	28 (19)	1 (0.6)	29 (9.6)	**0.001**
R1b	41 (27)	81 (54)	122 (40.6)	**0.042**
I2b1	17 (11)	4 (3)	21 (7)	**0.033**
L	5 (4)	1 (0.6)	6 (2)	0.099
R1a	5 (4)	7 (5)	11 (36)	0.560
I1	12 (8)	12 (8)	24 (8)	0.099
J2a1	5 (4)	4 (3)	9 (3)	0.741
I2a	1 (0.6)	4 (3)	5 (16)	0.181
E1b1b	1 (0.6)	23 (15)	24 (8)	**0.001**
T	1 (0.6)	7 (5)	8 (26)	**0.031**

Values represent the number of subjects, and percentages are in brackets. Analysis was performed using Fisher’s exact test with a significance level p<0.05; bold letters represent statistically significant differences.

Data were analyzed using logistic regression to estimate the probabilities of symptomatic CD condition related to haplogroups of Y chromosome using symptomatic patient ratio as the response variable, and if the patients carried the R1b haplogroup or not and if they were infected with *T. cruzi* or not as covariables. The multivariate analysis (Table 3) showed that CD patients who did not carry R1b (Rb-) had a 5.1 times higher likelihood of showing a cardio-thorax index greater than 0.5% than the Rb- nCD patients (OR = 5.1, 95% CI = 3.31-8.17). The patients identified to have the haplogroup R1b showed a significantly lower predictive value of developing cardiac symptoms diagnosed by thoracic XR. Taking into account the absence of genetic diversity between the study groups, we considered the entire population for the multivariate analysis. This analysis showed that chagasic and non-chagasic individuals who did not carry the haplogroup R1b were approximately 2.5 times more likely to present EcoCG with abnormalities than those who carried this haplogroup (OR = 2.50, 95% CI = 0.16-3.94) (Table 3).

**TABLE 3:** Multivariate logistic regression analysis of the R1b haplogroup.

Risk factor^a^	OR	95 % CI	P value
CD/nCD: cardio-thorax index higher than 0.5%			
R1b +	1.13	0.08-2.45	0.651
R1b -	5.10	3.31-8.17	0.001
All populations: EcoCG alterations			
R1b +	1.18	0.11-1.92	0.873
R1b -	2.50	0.16-3.94	0.001

^a^Adjusted for age, cholesterol level, and plasma lipoprotein level. **CD:** Chagas disease; **nCG:** Non-Chagas disease; **OR:** odd radio; **CI:** confidence interval.

## DISCUSSION

The fact that 20-30% of individuals infected with *T. cruzi* develop cardiomyopathy suggests the presence of a set of pathophysiological events that lead to the clinical manifestations of chagasic cardiomyopathy^18^. It is well known that the development of different cardiomyopathies (hypertrophic, dilated, idiopathic, chagasic) involves many genes, which means that the patient’s phenotype is generated by genetic interaction with the epigenetic variables^18^. We hypothesized that patients with cardiomyopathies may be associated with some Y chromosome haplogroups, which would increase or decrease the susceptibility to cardiac pathology by some factor.

In this study, we observed a high level of haplogroup diversity within the population, with the predominance of the haplogroups R1b and G2a. The former was more frequent in non-CD patients, while the latter was predominant in CD patients. To the best of our knowledge, this is the first study to demonstrate such a difference. Previous studies carried out in the Argentinean Mennonites community and the global population showed no difference in the frequency of Y chromosome haplogroups^19^ ^,^ ^20^, indicating that the results obtained in our work may be specific to a chagasic population. The identified haplogroups in the population in this study come from European descendants and were consistent with findings from previous studies^9^. The most common haplogroup among Western European inhabitants is the R1b of the Y chromosome, especially in areas near the Atlantic Ocean. The haplogroup G2a is present in 10% of the native European population. Haplogroup E1b1b is more common in speakers of Afro-Asian languages and individuals from Southern Europe, the Balkans, and most of sub-Saharan Africa^22^. In Argentina, the most common haplogroup in individuals from urban areas is the haplogroup R1b due to a large European migration wave that occurred in the previous century^21^ ^,^ ^22^. The most interesting finding from this study was that patients carrying haplogroup R1b presented with a lower number of heart alterations detected by thoracic XR and TTE. Similar studies have shown a potential role of genetic variations within the Y chromosome in determining cardiac health and sensitivity to cardiac conditions among men^23^. One of the strongest pieces of evidence underscoring this was the demonstration of the relationship between haplogroup I and the higher risk of coronary artery disease (CAD) in the British population^23^. Johansson et al. identified 12 pairs of genes sensitive to X-Y doses. 

Three of them were related to haplogroup I and participated in immunologic processes: UTY/UTX, PRKY/PRKX, and DM5D/KDM5C^24^. Eales et al. showed that the expression of *UTY* was reduced in blood samples from men with haplogroup I1 when compared with those with other haplogroups^25^. Khan et al. provided compelling evidence that Y chromosome lineage predicted T cell infiltration in key cardiovascular organs, which in turn may influence blood pressure^26^. In contrast, a recent study conducted in a young (under 18 years) male population showed that common variations in the Y chromosome were unlikely to play a role in cardiometabolic risk factors^27^. Different studies showed a relationship between Y chromosome haplogroups and the immune system and cardiovascular disease. Given that CD is infectious, combined studies on the Y chromosome’s role in immunologic pathway responses and the cardiac risk factors in men are necessary to explain the presence of different phenotypes of the cardiomyopathic disease. In line with this, Voskarides (2017) hypothesized that Y chromosome haplogroups may be used as additional indications for the risk of cardiovascular disease in men. However, this should strictly be a complementary test and should not, in any case, substitute the classical cardiovascular markers or examinations such as carotid artery screening, echocardiography, and lipoproteins profile^28^.

The present work was limited to a population consisting of middle-aged men, most of whom were undergoing pharmacological treatment, which may have potentially affected the phenotypic characteristics. Therefore, it may be difficult to generalize these findings to a wider healthy male population. It should be noted that due to the relatively low quantity of informative Y-SNP in the matrix Affymetrix 6.0, the study was limited only to the phylogenetic analysis of the main haplogroups. Further studies of the whole spectrum of the Y chromosome variations will be necessary to determine the possible role of the Y chromosome sequence in different human phenotypes.

On clinical assessment, we found that patients infected with *T. cruzi* **s**howed worse heart disease diagnoses than patients without infection. We observed that the Y chromosome may have significant effects on men’s cardiac health and may be considered to be a new risk factor to explain the different stages of CCC. Further studies should be carried out using a broader range of phenotypes to fully understand the biological bases of human health, men’s health, and CD^25^.
